# Epidemiological analysis of lower limb revascularization for peripheral arterial disease over 12 years on the public healthcare system in Brazil

**DOI:** 10.1590/1677-5449.202102152

**Published:** 2022-09-02

**Authors:** Nelson Wolosker, Marcelo Fiorelli Alexandrino da Silva, Maria Fernanda Cassino Portugal, Nickolas Stabellini, Antônio Eduardo Zerati, Claudia Szlejf, Edson Amaro, Marcelo Passos Teivelis

**Affiliations:** 1 Faculdade Israelita de Ciências da Saúde Albert Einstein, São Paulo, SP, Brasil.; 2 Universidade de São Paulo ­– USP, Faculdade de Medicina, São Paulo, SP, Brasil.; 3 Hospital Israelita Albert Einstein, São Paulo, SP, Brasil.

**Keywords:** big data, arteries, database, public health, vascular diseases, big data, artérias, saúde pública, doenças vasculares

## Abstract

**Background:**

Worldwide, peripheral arterial disease (PAD) is a disorder with high morbidity, affecting more than 200 million people.

**Objectives:**

Our objective was to analyze surgical treatment for PAD provided on the Brazilian Public Healthcare System over 12 years using publicly available data.

**Methods:**

The study was conducted with analysis of data available on the Brazilian Health Ministry’s database platform, assessing distributions of procedures and techniques over the years and their associated mortality and costs.

**Results:**

A total of 129,424 procedures were analyzed (performed either for claudication or critical ischemia, proportion unknown). The vast majority of procedures were endovascular (65.49%) and this disproportion exhibited a rising trend (p<0.001). There were 3,306 in-hospital deaths (mortality of 2.55%), with lower mortality in the endovascular group (1.2% vs. 5.0%, p=0.008). The overall governmental expenditure on these procedures was U$ 238,010,096.51, and endovascular procedures were on average significantly more expensive than open surgery (U$ 1,932.27 vs. U$ 1,517.32; p=0.016).

**Conclusions:**

Lower limb revascularizations were performed on the Brazilian Public Healthcare System with gradually increasing frequency from 2008 to 2019. Endovascular procedures were vastly more common and were associated with lower in-hospital mortality rates, but higher procedure costs.

## Resumo

**Contexto:**

A doença arterial periférica (DAP) é uma doença com alta morbidade global, afetando mais de 200 milhões de pessoas.

**Objetivos:**

Neste estudo, analisamos o tratamento cirúrgico para DAP no sistema público de saúde do Brasil no período de 12 anos, com base em dados publicamente disponíveis.

**Métodos:**

O estudo foi conduzido a partir da análise de dados disponíveis na plataforma do Departamento de Informática do Sistema Único de Saúde (DATASUS), do Ministério da Saúde, avaliando a distribuição da técnica cirúrgica utilizada, a mortalidade e o custo ao longo dos anos.

**Resultados:**

Um total de 129.424 procedimentos foram analisados (para claudicantes e isquemia crítica, em proporção desconhecida). A maiora dos procedimentos foi via endovascular (65,49%), com tendência de aumento nessa desproporção (p < 0,001). Houve 3.306 mortes intra-hospitalares (mortalidade de 2,55%) com menor mortalidade no grupo endovascular (1,2% vs. 5,0%; p = 0,008). O investimento governamental total para esses procedimentos foi de US$ 238.010.096,51, e os procedimentos endovasculares foram significativamente mais caros que a cirurgia aberta convencional (US$ 1.932,27 vs. US$ 1.517,32; p = 0,016).

**Conclusões:**

No sistema público de saúde brasileiro, as revascularizações de membros inferiores ocorreram com frequência crescente entre 2008 e 2019. Os procedimentos endovasculares foram mais comuns e relacionados a menor mortalidade intra-hospitalar, mas a maiores custos.

## INTRODUCTION

Estimates predict that over 20 million deaths will occur worldwide from cardiovascular diseases (CVD) by 2030 should current trends be maintained.^1^ The greatest burden is anticipated for low and middle-income countries (LMICs), mostly due to the expected population growth.^2^^,^^3^

Peripheral Arterial Disease (PAD), which is often marked by an abnormal ankle-brachial index,^4^^,^^5^ is a well-established predictor of cardiovascular mortality,^6^ and is estimated to affect over 200 million people worldwide.^7^ Over 40,000 yearly deaths were attributable to PAD according to the 2013 Global Burden of Disease Study, representing a 155% increase over 1990.^2^ Although diagnosis is simple and management is accessible and feasible, PAD remains relatively under-recognized and undertreated.^6^ Management should always include risk control with lifestyle changes and medical therapy and currently also comprises surgical or endovascular revascularization options, with a marked increase in the use of endovascular techniques over the past two decades.^8^

A comprehensive study by Gheorghe et al. aiming to determine the gaps in knowledge about the economic burden of CVD in LMICs identified a heterogeneous body of literature dominated by single-center retrospective cost studies conducted in secondary care settings.^9^ Specifically in Brazil, these authors reported finding only 8 papers offering economic estimates related to CVD.^9^ With regard to PAD, a Brazilian study published in 2016 revealed a high economic impact and a rising trend in use of endovascular techniques, with a 57% increase in endovascular procedures between 2008 and 2012.^10^ Another study, published by Wolosker et al., performed a descriptive analysis of treatment for PAD in the largest Brazilian city over the spam of 12 years, encompassing 10,951 procedures.^11^ These authors found that in the city of São Paulo endovascular procedures were more common than open surgeries and resulted in shorter hospital stays as well as lower perioperative mortality rates.^11^ However, no studies have been conducted to analyze management of PAD in the whole of Brazil.

Brazil’s population for the year 2020 was estimated at 211,755,692 inhabitants.^12^ Of these, as of June 2020, over 75% were entirely dependent on the Public Healthcare System (SUS), which is a tax-funded government service; whereas 24.1% had private supplementary healthcare coverage, whether financed individually or provided by an employer.^13^ In this scenario, this study aims to present the first descriptive analysis of the management of PAD in the whole of Brazil over the period from 2008 to 2019, assessing distributions of procedures and techniques over the years and their associated mortality and costs.

## METHODS

All information was extracted from the DATASUS portal, which is a digital platform on which the Brazilian government provides open data relating to procedures performed on the Public Healthcare System at accredited hospitals. Institutional accreditation with the system is a prerequisite for receiving government payments for the procedures performed. All data are appropriately de-identified.

This study was submitted to our institutional review board (protocol number 4324-20). Since all data were anonymous, an Informed Consent waiver was requested and granted.

Data on open surgical and endovascular procedures for treatment of PAD between 2008 and 2019 were selected from the platform along with information regarding the techniques used for procedures and associated mortality and costs. The values reported are the cost of hospitalization and include the surgical devices employed.

A total of 14 procedure codes were chosen to list procedures, selected from the SUS Procedures, Medicines, Ortheses, Prostheses, and Special Materials Table Management System (SIGTAP). Table 1 lists these procedures and codes and their respective frequencies by year.

**Table 1:** Number of lower limb revascularization procedures on the Brazilian Public Healthcare System by code and year from 2008 to 2019.

**Procedure**	**Procedure code**	**Number of cases by year**												
		**2008**	**2009**	**2010**	**2011**	**2012**	**2013**	**2014**	**2015**	**2016**	**2017**	**2018**	**2019**	**Total**
Axillobifemoral bypass	0406020310	46	41	50	50	45	68	37	39	36	34	43	25	514
Axillofemoral bypass	0406020329	39	25	42	53	45	26	39	37	40	44	21	24	435
Crossed femoro-femoral bypass	0406020345	332	387	397	317	290	284	260	244	292	263	260	230	3,556
Aorto-femoral thromboendarterectomy	0406020353	416	402	415	403	332	362	363	350	294	325	331	272	4,265
Aorto-iliac bypass/thromboendarterectomy	0406020361	169	145	123	125	129	90	92	101	120	121	98	74	1,387
Iliac-femoral bypass/thromboendarterectomy	0406020388	209	232	259	306	284	320	286	255	267	261	224	198	3,101
Distal artery revascularization bypass or thromboendarterectomy	0406020434	447	482	480	464	479	515	426	400	456	408	436	358	5,351
Distal artery revascularization bypass or thromboendarterectomy from the proximal femoro-popliteal segment	0406020442	1,405	1,373	1,304	1,274	1,206	1,132	1,101	1,006	1,116	1,086	1,126	925	14,054
Femoro-popliteal proximal bypass Revascularization or thromboendarterectomy	0406020450	1,532	1,283	1,163	1,096	1,103	968	879	893	819	832	758	675	12,001
Aortic intraluminal angioplasty, vena cava/iliac vessels (with stenting)	0406040028	621	857	873	772	773	1,113	1,204	1,275	1,197	1,187	1,307	1,150	12,329
Intraluminal aortic angioplasty, vena cava/iliac vessels (without stent)	0406040044	133	163	184	179	161	231	330	347	463	412	505	519	3,627
Intraluminal vessel angioplasty of distal artery (without stent)	0406040052	1,066	1,362	1,324	1,778	1,872	2,411	3,187	3,924	4,404	4,865	5,490	5,099	36,782
Intraluminal vessel angioplasty of distal artery (with uncovered stent)	0406040060	1,716	1,672	1,886	2,228	2,596	3,020	3,294	3,297	3,115	3,130	2,992	2,553	31,499
Reconstruction of the aortoiliac bifurcation with angioplasty and stenting	0406040281	26	21	38	41	44	46	46	54	40	55	75	37	523

Revascularization procedures were divided into two groups: Open Surgery and Endovascular Procedures. As is common with population code-based studies, miscoding or data loss may have occurred; considering the size of the sample, however, this is not expected to negatively impact the overall results.

All data were collected through an automated web scraping method. The codes employed were programmed in Python language (v. 2.7.13; Beaverton, OR, USA), using the Windows 10 Single Language operating system. Selection of fields on the DATASUS platform and posterior table adjustment were performed with Selenium WebDriver (v. 3.1.8; Selenium HQ, various contributors worldwide) and pandas packages (v. 2.7.13; Lambda Foundry, Inc. and PyData Development Team, NY, USA). All data were imported to Microsoft Office Excel 2016® (v. 16.0.4456.1003; Redmond, WA, USA) spread sheets after collection and treatment.

The monetary values in Brazilian Reais were converted into U.S. dollars using the exchange rate on December 31, 2012, which is the median date for the cases evaluated and was U$ 1 = R$ 2.04.

Statistical analysis was conducted as follows: the Chi-square test was used to identify trends in distribution of procedures and techniques over the years and groups were compared with regard to mortality rates and average costs using the Mann-Whitney test. The level of statistical significance adopted for all tests was 0.05.

## RESULTS

In total, 129,424 revascularization procedures for PAD were performed in Brazil on the Public Healthcare System from 2008 to 2019, with a yearly average of 10,785.33 procedures.

Considering this number and the approximate 160,722,570 inhabitants dependent on the Public Health System in Brazil in 2020, the procedure rate was 6.7 procedures per 100,000 inhabitants per year.

**Figure 1:**
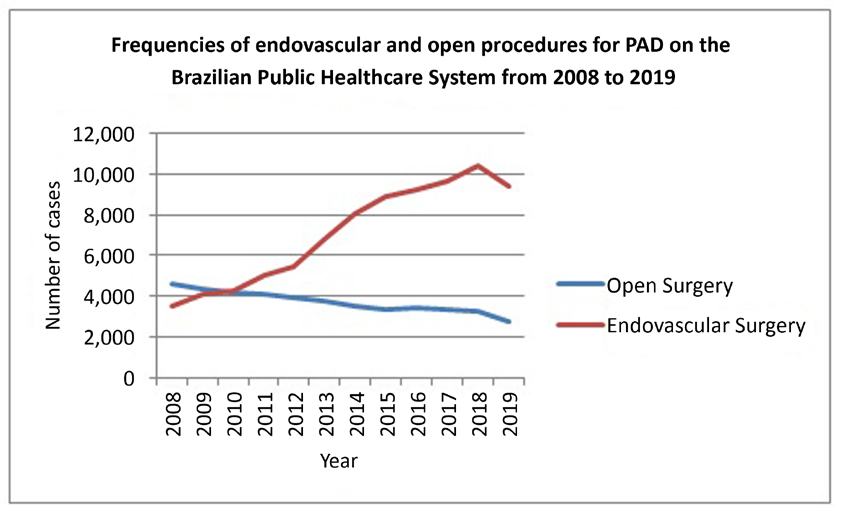
Frequencies of endovascular and open procedures for PAD on the Brazilian Public Healthcare System from 2008 to 2019.

Table 2 lists the frequencies of open and endovascular procedures performed over the years analyzed. The majority of procedures were conducted using endovascular techniques, with a technological transition observed especially from 2010 on, with a significant rising trend in the number of endovascular procedures, to the detriment of open surgery (*p*<0.001). The temporal trends of these findings are also illustrated in Figure 1.

**Table 2:** Absolute and relative frequency of endovascular and open procedures for PAD from 2008 to 2019.

	**Open Surgery**			**Endovascular Surgery**				***p*** ^*^
	**n**	**%**		**n**	**%**		**Total**	
2008	4,595	56.3		3,562	43.67		8,157	<0.001
2009	4,370	51.7		4,075	48.25		8,445	
2010	4,233	49.6		4,305	50.42		8,538	
2011	4,088	45.0		4,998	55.01		9,086	
2012	3,913	41.8		5,446	58.19		9,359	
2013	3,765	35.6		6,821	64.43		10,586	
2014	3,483	30.2		8,061	69.83		11,544	
2015	3,325	27.2		8,897	72.79		12,222	
2016	3,440	27.2		9,219	72.83		12,659	
2017	3,374	25.9		9,649	74.09		13,023	
2018	3,297	24.1		10,369	75.87		13,666	
2019	2,781	22.9		9,358	77.09		12,139	
**Total**	**44,664**	34.5		**84,760**	65.49		**129,424**	

* chi-square test for trend.

Mortality data are shown in Table 3. There were 3,306 in-hospital deaths (mortality rate of 2.55%). The in-hospital mortality rate was lower for endovascular procedures than for open surgery (1.2% *vs*. 5.0%, *p*=0.008).

**Table 3:** Absolute and relative mortality by geographic region and procedure type.

Geographic Region	Total				Procedure Type								
	Procedures	Mortality			Open Surgery					Endovascular Surgery			
		n	(%)		Procedures	Mortality n	(%)			Procedures	Mortality n	(%)	
North	1,978	62	3.13		560	34	6.07			1,418	28	1.97	
Northeast	19,799	447	2.26		4,455	256	5.75			15,344	191	1.24	
Southeast	56,821	1,650	2.90		22,446	1239	5.52			34,375	411	1.20	
South	45,689	976	2.14		15,032	624	4.15			30,657	352	1.15	
Midwest	5,137	171	3.33		2,171	104	4.79			2,966	67	2.26	
**Total**	**129,424**	**3,306**	**2.55**		**44,664**	**2,257**	**5.05**			**84,760**	**1,049**	**1.24**	
*p* ^*^											*0.008*		

* Mortality in the Open Surgery group was statistically higher than in the Endovascular Surgery group (p = 0.008), Mann-Whitney test.

Costs covered by the Public Healthcare System are presented in Table 4. The total expenditure on all procedures over the period evaluated was U$ 238,010,096.51. Endovascular procedures were significantly more expensive than open surgery (27.3% more expensive, U$ 1,932.27 *vs*. U$ 1,517.32; *p*=0.016).

**Table 4:** Sums paid by SUS, in US dollars, by geographic region and procedure type.

Geographic Region	Total Amount	Amount Paid per Procedure Type		Average Amount Paid per Patient	
		Endovascular Procedure	Open Surgery	Endovascular Surgery	Open Surgery
North	3,308,673.90	2,531,020.38	777,653.52	1,784.92	1,388.67
Northeast	31,738,677.58	24,998,691.02	6,739,986.55	1,629.22	1,512.90
Southeast	108,221,656.21	71,152,305.41	37,069,350.81	2,069.89	1,651.49
South	85,070,969.18	62,520,899.85	22,550,069.33	2,039.37	1,500.14
Midwest	9,670,119.64	6,341,132.13	3,328,987.51	2,137.94	1,533.39
**Total**	**238,010,096.51**	**167,544,048.79**	**70,466,047.72**	**1,932.27**	**1,517.32**
*p* ^*^				*0.016*	

* Cost per patient was statistically higher for endovascular surgery than open surgery (p =0.016), Mann-Whitney test.

## DISCUSSION

Our data demonstrate a gradual increase in the number of patients treated over the period evaluated, with an average of 10,785.33 procedures per year. There is a relative scarcity of information on the incidence of PAD worldwide.^14^ Moxey et al. reported a yearly average of 6,283.25 open revascularizations in England from 2002 to 2006 using Hospital Episode Statistics and Office for National Statistics data.^15^ Considering an estimated population of 50.97 million people in England in 2006,^16^ the rate of open procedures was 12.32 per 100,000 inhabitants, in contrast with 27.79 open procedures per 100,000 inhabitants in Brazil (80.52 per 100,000 when considering all PAD procedures together). In the UK, however, the incidence of PAD decreased steadily from 2000 to 2014, from 38.55 to 17.33 per 10,000 person-years,^17^ whereas results of a recent meta-analysis indicate that the prevalence of PAD increased from 2000 to 2010 in high-income countries.^14^ Long-term results from the German ‘getABI’ study, which enrolled patients aged 65 years and older, determined a PAD incidence of 203 per 10,000 person-years.^18^

In a study of French patients hospitalized for PAD under the National Health Insurance Scheme between 2007 and 2011, revascularizations were performed in 9.5% of cases, from a total of 7,266 patients evaluated.^19^ In our study, a high volume of treated patients and an increasing number of procedures per year suggests either an increase in disease incidence or an increment in government investment in the management of PAD.

In our sample, there was a clear predominance of endovascular procedures (65.49%), which was observed in all the years studied, except 2008 and 2009 (43.67 and 48.25%, respectively), indicating a technological transition around the year 2010. In the study by Wolosker et al., which focused only on the city of São Paulo, this technological transition with an inversion between the frequencies of open surgery and endovascular procedures seems to have happened earlier, even before 2008.^11^ In a study performed in a teaching hospital in São Paulo, the technological transition from endovascular procedures to open surgery was found to have begun as early as 2006.^20^

This expansion in the application of endovascular technique is in accordance with observations by other authors,^21^ and may be attributed to several reasons: the most obvious of which is, naturally, the relatively lower morbidity of endovascular procedures, which may widen the spectrum of treatable patients. Other authors suggest that a decrease in the threshold for intervention, with surgeries being performed for patients with claudication as well as those with critical limb ischemia, also represents a significant contribution to the increase in utilization of the endovascular technique.^22^^,^^23^ Some authors also propose that a rising awareness of the disease may be a contributing factor in the increased application of endovascular revascularization procedures.^24^ Finally, the fact that endovascular procedures may be less durable, thus requiring a number of reinterventions, may be a confounding factor when considering the frequency of techniques, especially because our de-identified dataset does not permit exclusion of readmissions, thereby limiting this evaluation. This database anonymity also prevents determination of the indications for surgery, whether for claudication or critical limb ischemia.

In-hospital mortality was higher for open surgery than for endovascular procedures (5.0% *vs.* 1.2%, *p*=0.008). These findings are in agreement with a recent meta-analysis of twenty-seven trials (seven randomized controlled trials and twenty retrospective trials) encompassing 17,536 patients, conducted by Tang et al., which found higher mortality during follow up (10.86% *vs.* 7.54%, p<0.05) in the open surgery group.^25^ The Bypass versus Angioplasty in Severe Ischemia of the Leg (BASIL) trial, which randomized 452 patients with severe limb ischemia due to infrainguinal disease to receive either a surgery-first or an angioplasty-first strategy, determined that at one-year open surgery and angioplasty did not differ significantly in amputation-free survival (71% bypass *vs*. 68% angioplasty; adjusted HR = 0.73, 95% CI [0.49–1.07]),^26^ but at 5-years follow-up, bypass surgery was associated with better overall survival (47% bypass *vs*. 41% angioplasty; adjusted HR = 0.61; 95% CI [0.50–0.75]; P < 0.009) and a non-significant difference in amputation free survival (38% bypass *vs*. 37% angioplasty; adjusted HR = 0.85; 95% CI [0.5–1.07]; P = 0.108).^27^

However, the mortality analysis in our sample is limited by two main factors: the fact that the indications for procedures cannot be established from the information available, so patients operated on for claudication cannot be separated from those with critical limb ischemia, which may have an impact on mortality rates; and the fact that the database anonymization makes patient follow-up impossible and all mortality data discussed refer exclusively to in-hospital deaths, so the analysis cannot contribute information with respect to long-term mortality rates.

In our sample, the government cost for endovascular procedures was higher than that for open surgery (U$ 1,932.27 *vs*. U$ 1,517.32; *p*=0.016). Studies focusing on the gross cost of hospitalization found that in general endovascular procedures involve shorter hospital stays and lower overall cost.^26^^,^^28^ The long-term economic assessment of the BASIL trial, however, showed that costs for open surgery were indeed higher in the first year ($34,378 for bypass *vs.* $25,909 for angioplasty), but that at the end of a 5-year follow-up this difference decreased ($45,322 for bypass *vs.* $39,801 for angioplasty) and was no longer significant, which the authors attributed to costs incurred subsequently because of the need for multiple reinterventions, including open surgery.^26^ It is important to mentioned, however, that the BASIL trial compared open surgery to plain angioplasty, and the bulk of literature lacks cost-analyses of newer endovascular options including atherectomy and drug eluting devices.^29^ Another important assertion with regard to this analysis in Brazil, and possibly other LMICs, is that in countries where most endovascular devices are not locally sourced, additional costs such as import taxes and duties may also apply.^30^

Although groups were stratified by regions for statistical purposes only, slight differences between Brazilian geographic regions were observed in both mortality rates and costs. These differences did not attain statistical significance (*p* = 0.822 for mortality rates and *p*=0.663 for costs, Kruskall-Wallis test). We attribute the differences to the occurrence of different procedures in different years in each region.

From the published data available, it can be surmised that the Brazilian Public Health System reimburses procedures equally, regardless of where they are performed. What may differ is the actual cost to the operating institution of each procedure. However, these data are not publicly available. Therefore, the number and type of procedures performed each year in each region will result in differences in the average value paid per procedure, although national reimbursement rates are the same.

### Limitations

All data in this study were obtained from publicly available websites using web scraping code. Manual collection of this volume of data would be rather laborious, although technically feasible. On the other hand, automated collection enables information to be acquired in a faster and less complicated fashion. It should be acknowledged, however, that this process is not free from coding errors, especially considering that all information is sourced from an administrative database rather than from medical records, as well as the fact that the coding system may permit a certain ambiguity of codes for procedures employed for different diseases, such as revascularizations for PAD and because of trauma. Since diagnoses cannot be tracked from the de-identified database, exclusion of unwanted cases is impossible; but it may be surmised from the large numbers of cases that a very small proportion of the information is incorrect. Unfortunately, unlike the municipal platforms, the nationwide platform does not permit exclusion of disease codes, which precludes exclusion of trauma cases (International Classification of Diseases 10th revision – ICD-10 – S or T) that undergo revascularization. In order to address this issue, the proportion of cases subjected to revascularization under ICD-10 diagnostics S or T – that is, patients treated for trauma, instead of PAD – was evaluated for the city of São Paulo, which is Brazil’s largest urban center, using the Wolosker patient sample.^11^ From 2008 to 2018, only 0.14% of all revascularizations in the São Paulo data were classified as being a consequence of trauma. Thus, if this proportion can be extrapolated to the whole of Brazil by analogy, it could be assumed that the number of non-excludable trauma cases in this sample is of little relevance.

As is inherent to retrospective analyses, our study is limited by loss of patient information and possible miscoding of the administrative database. Because only hospitals duly accredited on the SIGTAP register can populate the database, information will certainly have been lost from centers without accreditation that may have performed procedures in urgent scenarios.

The deidentified nature of the public dataset also precludes collection of demographic data such as patients’ age and sex, as well as more accurate follow-up.

Lastly, we are unable to provide input related to the need for reinterventions and the weight they bear on the frequency of endovascular procedures.

In spite of its limitations, this is a comprehensive analysis of the public healthcare system’s management of PAD in one of the world’s largest and most populous LMICs, encompassing a 12-year interval and over 120,000 procedures, yielding a representative assessment of a real-world sample of PAD patients. This study also constitutes a useful tool to improve understanding of the public system and to guide allocation of healthcare funding.

## CONCLUSIONS

The number of patients treated for PAD with surgical management by the Brazilian Public Healthcare System increased gradually in the period between 2008 and 2019. Endovascular procedures were vastly more common, especially after a technological transition occurring in 2010. Endovascular procedures were associated with lower in-hospital mortality rates, at the expense of higher procedural costs (27.3% higher).
